# Correlation between oral microbiome characteristics and clinical phenotypes in patients with GERD and its changes after anti-reflux surgery

**DOI:** 10.3389/fmicb.2026.1878947

**Published:** 2026-08-17

**Authors:** ZhongYu Wang, Yu Liu, Zhe Han, Fan-Ke Wang

**Affiliations:** 1 Gastrointestinal Disease Center, The First Hospital of Hebei Medical University, Shijiazhuang, Hebei, China; 2 Hebei Medical University, Shijiazhuang, Hebei, China; 3 Hebei University, Baoding, Hebei, China; 4 Department of Breast Surgery, Hengshui People's Hospital, Hengshui, Hebei, China

**Keywords:** anti-reflux surgery, clinical phenotypes, gastroesophageal reflux disease, laparoscopic fundoplication, oral microbiome

## Abstract

**Background:**

Gastroesophageal reflux disease is defined by the reflux of gastroduodenal material into the esophagus, leading to troublesome symptoms and complications. Although the gastrointestinal microbiome has been increasingly implicated in gastroesophageal reflux disease pathogenesis, existing research has largely centered on esophageal and intestinal microbiota. The alterations and clinical relevance of the oral microbiome in gastroesophageal reflux disease remain underexplored. Therefore, this study was designed to: (1) systematically compare the oral microbiome structure between gastroesophageal reflux disease patients and healthy controls, and to examine the correlations between differentially abundant microbial taxa and key clinical phenotypes; (2) longitudinally assess the dynamic changes in the oral microbiome after anti-reflux surgery.

**Methods:**

We conducted a study integrating a case-control design with a longitudinal self-controlled component. Initially, 40 patients and 20 healthy volunteers were enrolled. Following exclusions based on diagnostic confirmation, 36 gastroesophageal reflux disease patients comprised the preoperative group (Group Pre), and 17 age-, gender-, and BMI-matched healthy volunteers served as controls (Group HC). Non-stimulated whole saliva samples were collected from all participants. A subgroup of 18 patients from Group Pre subsequently underwent laparoscopic fundoplication. Their saliva samples collected 3 months postoperatively constituted the postoperative group (Group Post) for longitudinal comparison. The V3–V4 hypervariable regions of the bacterial 16S rRNA gene were amplified and sequenced using high-throughput sequencing. Sequencing data were processed and analyzed with bioinformatics pipelines to evaluate α- and β-diversity. Differential microbial features across groups were identified using Linear Discriminant Analysis Effect Size. Correlations between microbial relative abundance and clinical parameters were assessed *via* Spearman's rank correlation. Changes in the microbiome following surgery were evaluated using paired non-parametric statistical tests.

**Results:**

(1) Cross-sectional comparisons: no significant differences were observed in α-diversity indices (ACE, Chao1, Shannon, and Simpson; all *P* > 0.05) of the oral microbiome between GERD patients (Group Pre) and healthy controls (Group HC). In contrast, β-diversity analysis indicated a significant overall structural disparity between the groups (PERMANOVA, *R*^2^ = 0.044, *P* = 0.003). Linear Discriminant Analysis Effect Size (LEfSe) identified discriminant taxa distinguishing the two groups, with genera like *Alloprevotella* and *Veillonella* being enriched in Group Pre, and *Streptococcus* enriched in Group HC. Furthermore, Spearman correlation analyses identified significant associations between specific genera and clinical parameters: the relative abundance of *Veillonella* showed a positive correlation with the gastroesophageal reflux disease questionaire score (*r* = 0.347, *P* = 0.038), and that of *Alloprevotella* correlated positively with the severity of esophagitis as graded by the Los Angeles classification (*r* = 0.335, *P* = 0.046).

(2) Longitudinal analysis revealed that bacterial genera such as *Veillonella*, which were associated with phenotypes in cross-sectional studies, did not show significant changes in the oral microbiota between the Pre- and Post- groups. The most characteristic alteration was that the relative abundance of the pro-inflammatory genus *Fusobacterium*was succeeded by *Selenomonas*, a genus with potential metabolic benefits.

**Conclusion:**

Our study demonstrates that gastroesophageal reflux disease is characterized by a specific oral dysbiosis, with the abundance of particular bacterial taxa correlating with clinical disease severity. Although laparoscopic anti-reflux surgery did not drastically alter the global oral microbiome architecture, it induced a targeted ecological shift. This shift was marked by the suppression of pro-inflammatory taxa, an increase in potentially beneficial commensals, and a trend toward restored α-diversity. These findings suggest that laparoscopic anti-reflux surgery may facilitate a shift of the oral microbiome toward a healthier ecological state following restoration of the anti-reflux barrier function. This observation provides a new microbiological perspective for understanding the broader physiological impact of anti-reflux surgery.

## 1 Introduction

Gastroesophageal reflux disease (GERD) ranks among the most prevalent digestive disorders globally, affecting 10%−20% of the population in Western countries. In China, its prevalence is rising steadily, driven by an aging population, increasing obesity rates, and improved diagnostic capabilities, with approximately 2.5%−7.8% of individuals reporting typical symptoms (El-Serag et al., 2014; Fock et al., 2016). The hallmark symptoms of heartburn and regurgitation substantially impair quality of life and can lead to serious complications, including esophagitis, Barrett's esophagus, and esophageal adenocarcinoma. The pathogenesis of GERD is multifactorial, involving lower esophageal sphincter (LES) dysfunction, compromised esophageal mucosal defense, impaired esophageal clearance, and anatomical defects like hiatal hernia (Dunbar, 2024).

Clinically, GERD is highly heterogeneous. Patients present with a broad spectrum of manifestations, including varying symptom profiles, degrees of pathological acid exposure, and endoscopic findings ranging from non-erosive reflux disease to erosive esophagitis and Barrett's esophagus (Kahrilas, 2003). This phenotypic diversity suggests that mechanisms beyond traditional acid-motor dysfunction are likely involved in disease pathogenesis and progression.

The advent of microbiome science has recently shed light on the potential role of gastrointestinal microbes in GERD. The human gut microbiota, now recognized as a virtual metabolic organ, significantly influences host health and disease by modulating immunity, metabolism, and mucosal barrier integrity (Guan et al., 2025). Initial research has primarily focused on the esophageal and gut microbiota in GERD. Studies report alterations in the esophageal microbiome of GERD and Barrett's esophagus patients, such as an increased abundance of Gram-negative bacteria, while the gut microbiota may influence GERD indirectly *via* axes like the gut-lung and gut-brain pathways (Enaud et al., 2020; Yang et al., 2009; Zheng and Tao, 2025). In contrast, the oral microbiome—the second most diverse microbial community in the human body and the gateway to the gut—has been largely overlooked in GERD research. Given the anatomical continuity between the oral cavity and esophagus, microbial exchange is plausible through swallowing and reflux events. It is hypothesized that refluxate may transport oral bacteria distally or alter the esophageal niche to favor specific colonization. Conversely, chronic reflux may modify the oral environment, potentially selecting for a distinct oral microbial community (Wang et al., 2020). However, systematic evidence is lacking regarding whether a characteristic oral dysbiosis exists in GERD and whether it correlates with clinical disease phenotypes.

Although increasing attention has recently been directed toward the oral microbiome in GERD and other upper gastrointestinal diseases, current evidence remains limited and heterogeneous. Previous studies have demonstrated that patients with reflux esophagitis or Barrett's esophagus exhibit alterations in salivary microbial composition compared with healthy individuals, with changes involving genera such as *Veillonella, Prevotella, Streptococcus*, and *Neisseria*. These findings suggest that the oral cavity may reflect pathological processes occurring along the upper digestive tract. However, most published studies have been exploratory, based on relatively small sample sizes, and primarily focused on describing taxonomic differences between patients and controls. Few investigations have evaluated the relationship between oral microbial alterations and objective clinical phenotypes, including symptom severity, esophageal acid exposure, and endoscopic mucosal injury. Furthermore, current oral microbiome studies in GERD have almost exclusively adopted cross-sectional designs, providing only a static snapshot of microbial composition. Whether successful restoration of the anti-reflux barrier through laparoscopic fundoplication results in longitudinal remodeling of the oral microbiome has not previously been investigated. Consequently, it remains unclear whether the observed microbial alterations simply accompany GERD or dynamically respond to therapeutic intervention. Addressing these knowledge gaps is essential for clarifying the clinical significance of oral microbial dysbiosis and for determining its potential value as a non-invasive biomarker for disease monitoring and treatment evaluation. Therefore, the present study was designed using a combined cross-sectional and longitudinal approach to comprehensively characterize oral microbiome alterations in GERD, investigate their associations with clinically relevant disease phenotypes, and evaluate the ecological changes occurring after anti-reflux surgery. By integrating microbial profiling with objective clinical indicators and postoperative follow-up, this study extends current knowledge beyond descriptive taxonomic comparisons and provides new insights into the potential clinical relevance of the oral microbiome in GERD.

Proton pump inhibitors (PPIs) remain the first-line medical therapy for GERD, yet limitations include incomplete response in a subset of patients and concerns over long-term use (Fass et al., 2005). For patients with severe, refractory, or complicated GERD, laparoscopic fundoplication (LF) is the gold-standard surgical intervention, providing durable restoration of the anti-reflux barrier and symptom control (Lee et al., 2023; Targarona et al., 2004). While surgical success is traditionally gauged by symptomatic and pH-metric outcomes, its potential impact on the microbial dysbiosis associated with GERD is unknown. Specifically, it remains unclear whether surgical restoration of the anti-reflux barrier may indirectly influence the oral microbial ecosystem by reducing chronic exposure of the upper aerodigestive tract to refluxate. Investigating the longitudinal effect of surgery on the microbiome may offer novel insights into its mechanisms of action and uncover potential microbial biomarkers for therapeutic response.

Therefore, we hypothesize that: (1) GERD is associated with a distinct oral microbiome signature that correlates with key clinical phenotypes (symptom severity, acid exposure, and mucosal injury); (2) Anti-reflux surgery may be associated with ecological remodeling of the oral microbiome following anatomical and functional restoration of the anti-reflux barrier.

To test these hypotheses, this study aimed to: (1) compare the oral microbiome structure and composition between GERD patients and healthy controls, and analyze associations between differential microbial features and clinical indices (GERDQ, DeMeester score, Los Angeles grade); and (2) longitudinally assess the dynamic changes in the oral microbiome before and after LF to evaluate the procedure's modulatory effect. Our findings may provide a new microbiological perspective on GERD pathogenesis and the systemic impact of its surgical treatment.

## 2 Materials and methods

### 2.1 Study subjects and grouping

A total of 40 patients diagnosed with GERD and scheduled for LF at the Second Department of Gastroenterology, The First Hospital of Hebei Medical University, between September 2024 and September 2025, were prospectively enrolled as the patient group (Group Pre). Concurrently, 20 healthy volunteers were recruited as the control group (Group HC).

*Inclusion criteria for group pre were:* (1) Age between 18 and 75 years; (2) Meeting the diagnostic criteria for GERD and satisfying at least one of the following conditions: (i) Failed medical management, including unsatisfactory symptom control, severe symptoms refractory to acid suppression, or intolerable side effects; (ii) Effective but requiring long-term maintenance therapy, with unwillingness for prolonged medication or concerns over its burden; (iii) Presence of GERD complications, such as Barrett's esophagus (BE), esophageal stricture, ulcer, or esophagitis graded LA-B or above; (iv) Chronic or recurrent extra-esophageal symptoms/complications, including reflux-related asthma, cough, laryngopharyngeal symptoms, laryngospasm, and aspiration; (3) No history of major cardiopulmonary diseases, and fit for anti-reflux surgery under general anesthesia with intubation.

*Inclusion criteria for group HC were:* (1) Age between 18 and 75 years; (2) No history of heartburn or regurgitation.

*Exclusion criteria for both groups were:* (1) Diagnosis of oral, esophageal, or gastric cancer; (2) History of upper gastrointestinal surgery; (3) Participants who had received systemic antibiotics within 7 days before either baseline or postoperative saliva collection were excluded from microbiome analysis; (4) Refusal to provide a saliva sample.

This study was approved by the Medical Ethics Committee of The First Hospital of Hebei Medical University. Written informed consent was obtained from all participants.

### 2.2 Study protocol

Baseline data, including name, sex, age, height, weight, history of smoking, alcohol consumption, hypertension, and diabetes, were collected from all enrolled subjects. All participants completed the Gastroesophageal Reflux Disease Questionnaire. Additionally, for patients in Group Pre, the DeMeester score from 24-h pH monitoring and the LA classification from gastroscopy were recorded.

Saliva samples were collected from all subjects between 7:00 and 9:00 AM after an overnight fast and prior to toothbrushing. Participants were instructed to refrain from tooth brushing, mouthwash use, chewing gum, smoking, and food or beverage intake before saliva collection. Under the guidance of researchers, participants provided approximately 1.5 ml of unstimulated, naturally flowing saliva into sterile saliva collection tubes. Collected samples were immediately placed in liquid nitrogen, swiftly transferred, and stored at −80 C until DNA extraction. For the 18 GERD patients who successfully underwent LF, saliva samples were collected again using the identical protocol at the 3-month postoperative follow-up visit.

Before surgery, patients received individualized proton pump inhibitor therapy according to routine clinical practice. Medication type and dosage were determined by the treating physicians and were not standardized because pharmacological treatment was not an outcome of the present study.

Routine PPI therapy was not prescribed after successful fundoplication. At the 3-month saliva collection, patients were not receiving regular anti-reflux medication unless clinically indicated.

#### 2.2.1 Gastroesophageal reflux disease questionnaire

The GERDQ is a brief, self-administered instrument widely used internationally for screening, aiding diagnosis, and preliminary assessment of the disease impact in GERD. Its validity has been confirmed in multiple studies (Jonasson et al., 2013). The questionnaire consists of 6 items (Table 1): heartburn, regurgitation, epigastric pain, nausea, sleep disturbance due to heartburn/regurgitation, and use of over-the-counter medications for symptom relief.

**Table 1:** Gastroesophageal reflux disease questionnaire.

Over the past 7 days, how often did you experience the following?	Points			
	0 points	1 points	2 points	3 points
1. heartburn	Never	1 day	2–3 days	4–7 days
2. regurgitation	Never	1 day	2–3 days	4–7 days
3. pain in the center of the upper stomach	4–7 days	2–3 days	1 day	0 day
4. nausea	4–7 days	2–3 days	1 day	0 day
5. can't get a good night's sleep because of heartburn and/or regurgitation	Never	1 day	2–3 days	4–7 days
6. take additional medication for heartburn and/or regurgitation other than what the physician told	Never	1 day	2–3 days	4–7 days

#### 2.2.2 Gastroscopic examination

Gastroscopy is the gold standard method for evaluating the upper gastrointestinal mucosa, determining the severity of esophagitis, identifying complications, and excluding other malignant lesions in patients with GERD. The Los Angeles (LA) classification is an endoscopic grading system for reflux esophagitis. Based on the length of mucosal breaks, their confluence, and circumferential extent, it categorizes reflux esophagitis into four grades of increasing severity: A, B, C, and D (Table 2).

**Table 2:** Los Angeles classification of esophagitis.

Grade A	One or more mucosal breaks ≤ 5 mm in length, confined to the mucosal folds, and not extending between the tops of two mucosal folds
Grade B	One or more mucosal breaks >5 mm in length, confined to the mucosal folds, and not extending between the tops of two mucosal folds
Grade C	Mucosal breaks that extend continuously between the tops of two or more mucosal folds but involve < 75% of the esophageal circumference
Grade D	Mucosal breaks that involve ≥75% of the esophageal circumference

#### 2.2.3 24-h impedance-pH monitoring

24-h multichannel intraliminal impedance-pH monitoring is currently the gold standard for the diagnosis of GERD. By simultaneously monitoring pH and impedance changes within the esophagus, it can detect not only acid reflux but also weakly acidic and non-acid reflux events, and can clarify the association between reflux episodes and symptoms. Prior to the examination, patients were required to discontinue proton pump inhibitors for at least 1 week, H2 receptor antagonists for at least 3 days, and prokinetic agents. A thin, flexible catheter equipped with pH and impedance sensors was transnasally placed into the esophagus. The proximal end of the catheter was connected to a portable data recorder. Patients recorded the timings of meals, recumbent periods, and the precise onset of any symptoms during the 24-h monitoring period.

Key parameters derived from the 24-h MII-pH monitoring included the DeMeester score, the number of acid reflux episodes, weakly acidic reflux episodes, non-acid reflux episodes, and the symptom index.

#### 2.2.4 Saliva sample collection and processing

Each enrolled subject was provided with a saliva collection tube. They were instructed to collect unstimulated saliva in the early morning after an overnight fast and before toothbrushing. The specific procedure was as follows: subjects were asked to keep their mouth slightly open, gently rest the tip of the tongue against the hard palate, and tilt the head slightly forward, allowing saliva to flow naturally into the collection tube until a volume of approximately 1.5 ml was obtained. Immediately after collection, the sample was stored in a −80 °C temperature freezer.

#### 2.2.5 Surgical procedure

The procedure was performed under general anesthesia with endotracheal intubation. After establishing pneumoperitoneum, a laparoscope and instruments were introduced. The left lobe of the liver was retracted to expose the operative field. The gastrohepatic ligament was divided, and the peritoneum over the right crus and the anterior aspect of the esophagus was dissected. The gastrophrenic and esophagophrenic ligaments were subsequently divided, exposing the left crus. The esophagus was mobilized circumferentially to achieve an adequate intra-abdominal length of at least 5 cm, creating the retroesophageal window. Any hiatal hernia contents were reduced. The diaphragmatic crura were approximated posterior to the esophagus with interrupted sutures. For hiatal defects larger than 5 cm, a mesh reinforcement was considered. Subsequently, a laparoscopic fundoplication was performed. The gastric fundus was wrapped around the distal esophagus. The type of fundoplication was selected based on intraoperative assessment. The fundoplication was secured with interrupted sutures to create a tension-free wrap around the lower esophagus. The standardized seven-step procedure for laparoscopic hiatal hernia repair was referenced for technical details (Taicheng et al., 2025).

#### 2.2.6 Statistical analysis

Data analysis and graphical representation were performed using SPSS 27.0 and QIIME2 software. Measurement data conforming to a normal distribution are presented as the mean ± standard deviation, and comparisons between groups were conducted using the Student's *t*-test. Data not conforming to a normal distribution are expressed as the median, with the Mann-Whitney U test or the Kruskal-Wallis H test employed for between-group comparisons. Count data are presented as numbers, and the chi-square test was used for comparisons. Differentially abundant bacterial taxa between groups were identified using LEfSe analysis, with a threshold of LDA Score >4.0 and a *P*-value < 0.05. Spearman's rank correlation analysis was applied to assess the associations between microbial taxa and clinical parameters. Longitudinal comparisons before and after surgery were performed using the Wilcoxon signed-rank test. All statistical tests were two-sided, and a *P*-value < 0.05 was considered statistically significant.

## 3 Results

### 3.1 Baseline characteristics of the study population

There were no statistically significant differences (*P* > 0.05) in baseline characteristics, including age, sex, BMI, history of smoking, alcohol consumption, hypertension, and diabetes, between Group Pre and Group HC, indicating good comparability between the two groups. The GERDQ score was significantly higher in Group Pre than in the normal range (Table 3).

**Table 3:** Baseline characteristics of the study participants.

Variable	Group pre (*n* = 36)	Group HC (*n* = 17)	*P*-value
Male	61.1%	70.6%	0.502
Age (years)	53.72 ± 10.69	48.35 ± 11.18	0.099
BMI (kg/m2)	24.06 ± 3.56	22.60 ± 3.05	0.153
Smoking (%)	16.7%	11.8%	0.645
Alcohol drinking (%)	8.3%	17.6%	0.131
GERDQ (score)	10.17 ± 2.89	4.94 ± 0.90	< 0.01

### 3.2 Differences in oral microbiome structure between group pre and group HC

#### 3.2.1 Alpha-diversity analysis

**Figure 1:**
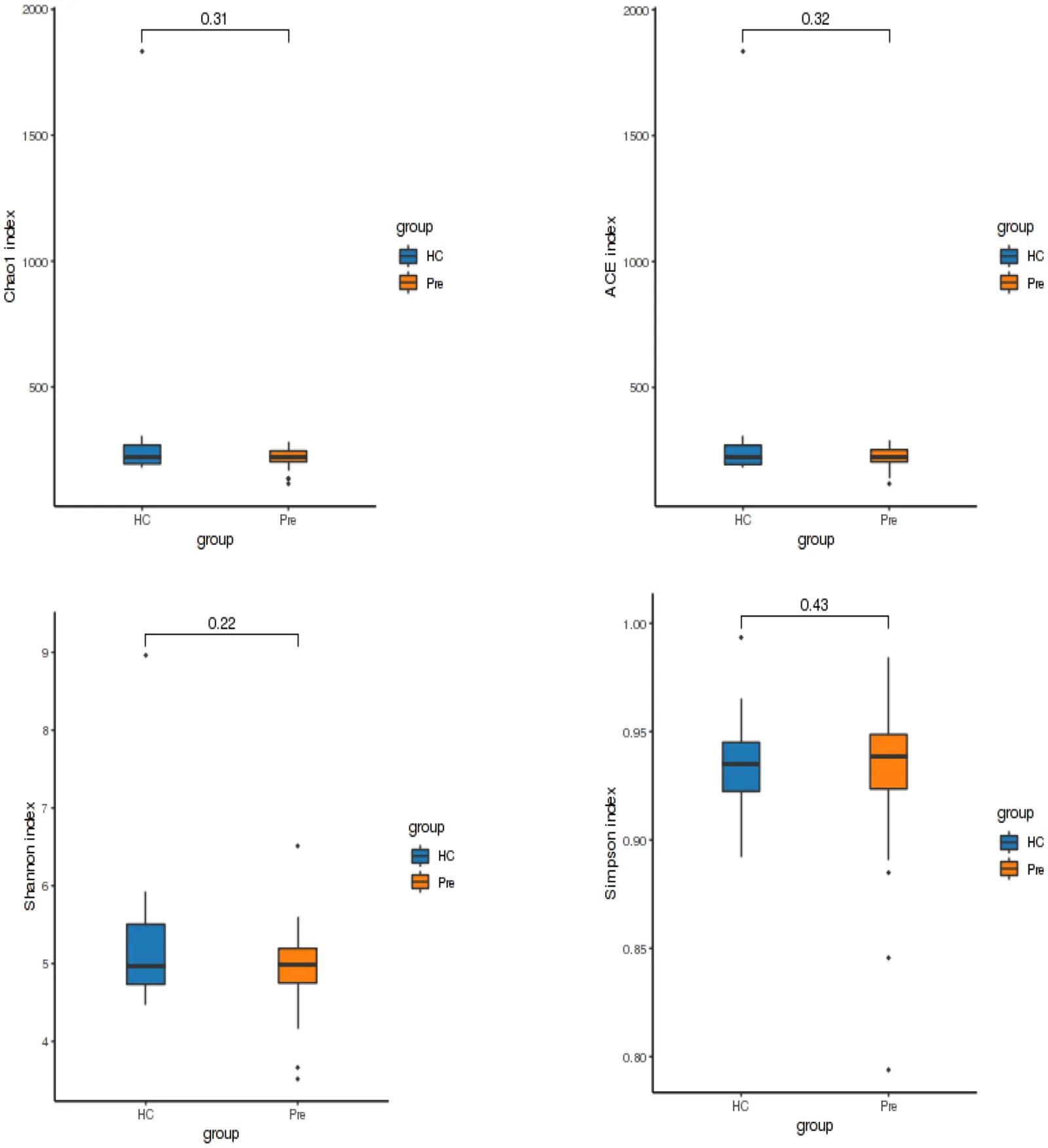
Comparative analysis of alpha diversity between the preoperative pre group and the HC group.

No significant differences were observed in the ACE, Chao1, Shannon, and Simpson indices between the two groups (all *P* > 0.05), indicating that the species richness and evenness of the salivary microbiota were similar between Group Pre and Group HC (Figure 1).

#### 3.2.2 Beta-diversity analysis

**Figure 2:**
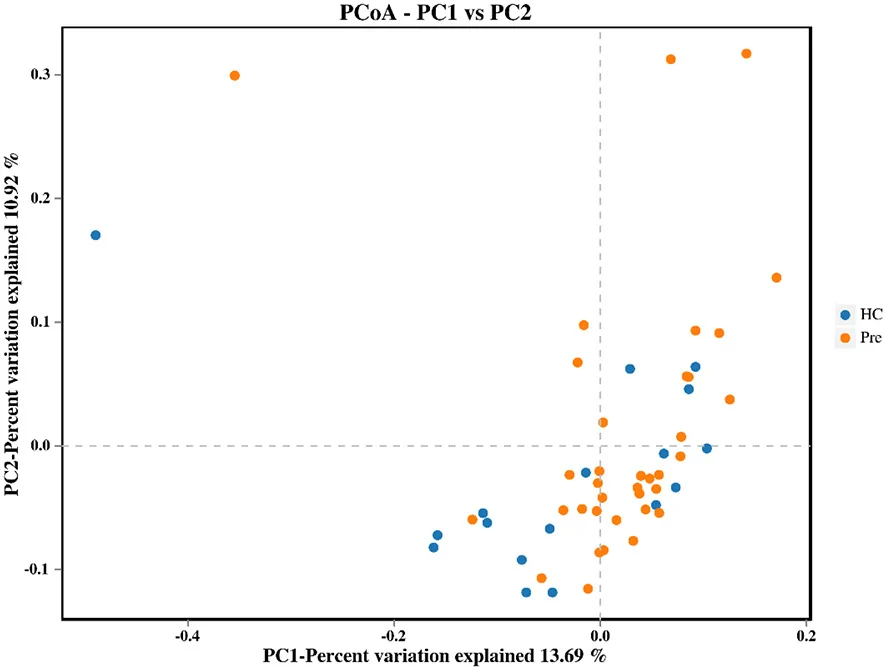
P-CoA analysis between the pre group and the HC group.

**Figure 3:**
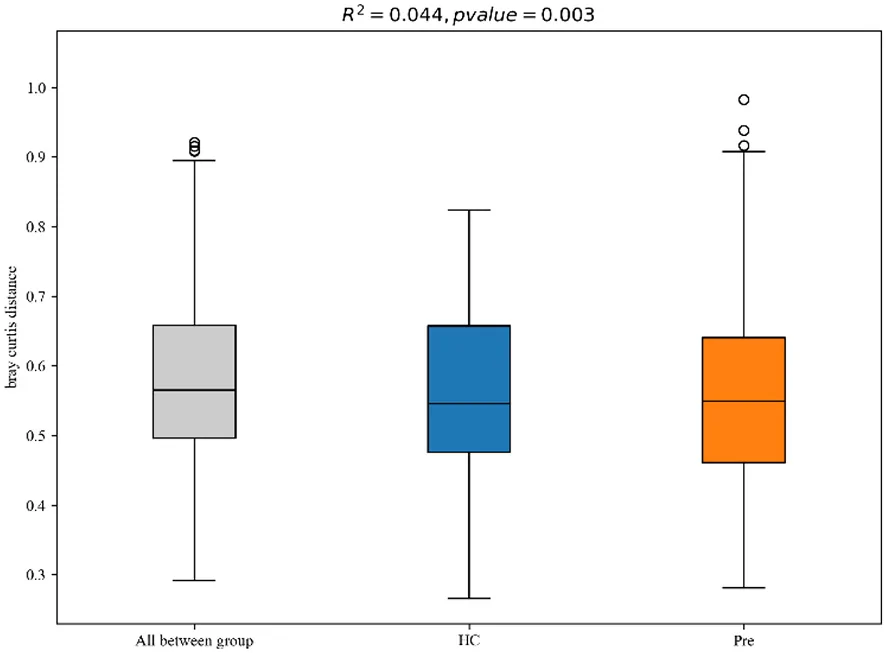
Comparative analysis of PERMANOVA between the pre group and the HC group.

Principal Coordinates Analysis (P-CoA) based on Bray–Curtis distance demonstrated partial overlap between Group Pre and Group HC, although a subtle shift in overall community composition could still be observed between groups (Figure 2). PERMANOVA analysis further indicated that this compositional difference was statistically significant (*R*^2^ = 0.044, *P* = 0.003; Figure 3). However, the relatively small effect size suggests that the magnitude of between-group separation was modest.

#### 3.2.3 Microbial composition and differential abundance analysis

**Figure 4:**
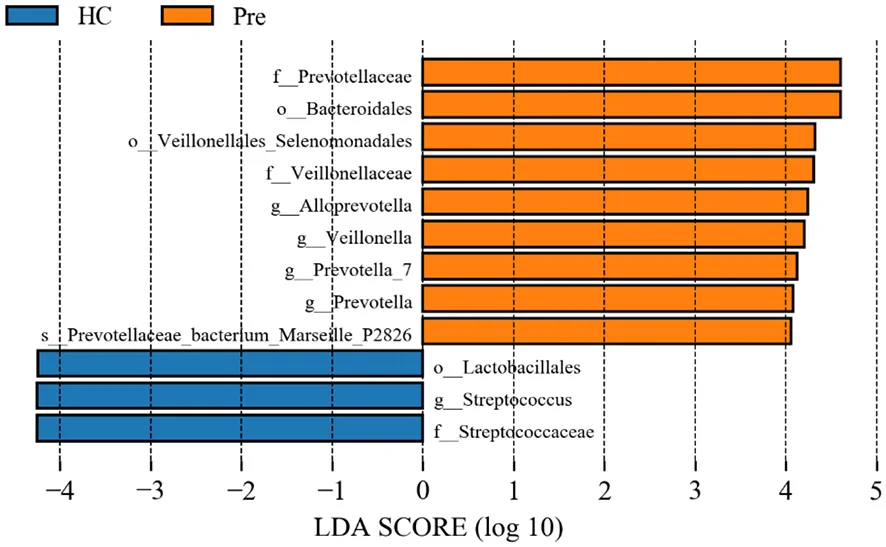
LEfSe analysis between the pre group and the HC group.

To identify statistically significant biomarker taxa distinguishing the two groups, LEfSe was performed with a threshold of LDA score >4. The results indicated that Group Pre was enriched, compared to Group HC, in the order *Bacteroidales* and the order *Veillonellales_Selenomonadales*, the family *Alloprevotellaceae* and the family *Veillonellaceae*, as well as the genus *Veillonella* and the genus *Alloprevotella*. In contrast, the genus *Streptococcus* was significantly more abundant in Group HC than in Group Pre (Figure 4).

### 3.3 Correlation between differential oral microbiota and clinical phenotypes

To explore potential associations between the oral microbiota and clinical phenotypes of GERD, Spearman's rank correlation analysis was performed between the relative abundances of key bacterial genera and clinical indices. As shown in Figure 4, the core clinical indices of GERD exhibited the expected significant positive correlations: symptom burden (GERDQ score) was significantly positively correlated with esophageal acid exposure (DeMeester score; *r* = 0.440, *P* = 0.007), and the DeMeester score, in turn, showed a strong positive correlation with the severity of mucosal injury (Los Angeles classification; *r* = 0.591, *P* < 0.001).

Regarding microbiota-phenotype associations (Table 4), the analysis revealed specific correlation patterns. The relative abundance of *Veillonella* was significantly positively correlated with the GERDQ score (*r* = 0.347, *P* = 0.038), suggesting that enrichment of this genus is associated with more severe reflux symptoms. In contrast, the relative abundance of *Alloprevotella* showed a significant positive correlation with the degree of esophageal mucosal injury (Los Angeles classification; *r* = 0.335, *P* = 0.046), indicating its potential link to the process of reflux-induced tissue damage. Notably, the abundance of *Streptococcus*, a core commensal genus in the oral cavity, was not significantly correlated with any of the included clinical indices (all *P* > 0.05).

**Table 4:** Spearman's rank correlation analysis.

Genus	Measure	GERDQ score	DeMeester score	Los Angeles classification
*Veillonella*	Correlation coefficient	0.347^*^	0.074	0.130
	*P*-value	0.038	0.667	0.451
*Alloprevotella*	Correlation coefficient	0.055	0.079	0.355^*^
	*P*-value	0.748	0.646	0.046
*Streptococcus*	Correlation coefficient	−0.062	−0.016	0.076
	*P*-value	0.721	0.928	0.661

^*^Indicates a statistically significant correlation (*P* < 0.05).

### 3.4 Longitudinal changes in oral microbiome after anti-reflux surgery

#### 3.4.1 Clinical efficacy of anti-reflux surgery

First, paired comparisons of clinical indices were performed for the 18 patients who underwent laparoscopic fundoplication, before and after surgery. As shown in Table 5, at 3 months postoperatively, the mean GERDQ score significantly decreased from the preoperative value of 10.25 to 6.13 (*P* < 0.05).

**Table 5:** Comparison of reflux-related outcomes pre- and post- surgery.

Variables assessed	GERDQ score
Pre to surgery	10.25 ± 3.19
Post to surgery	6.13 ± 2.58
*P-*value	< 0.01

#### 3.4.2 Changes in alpha-diversity

**Figure 5:**
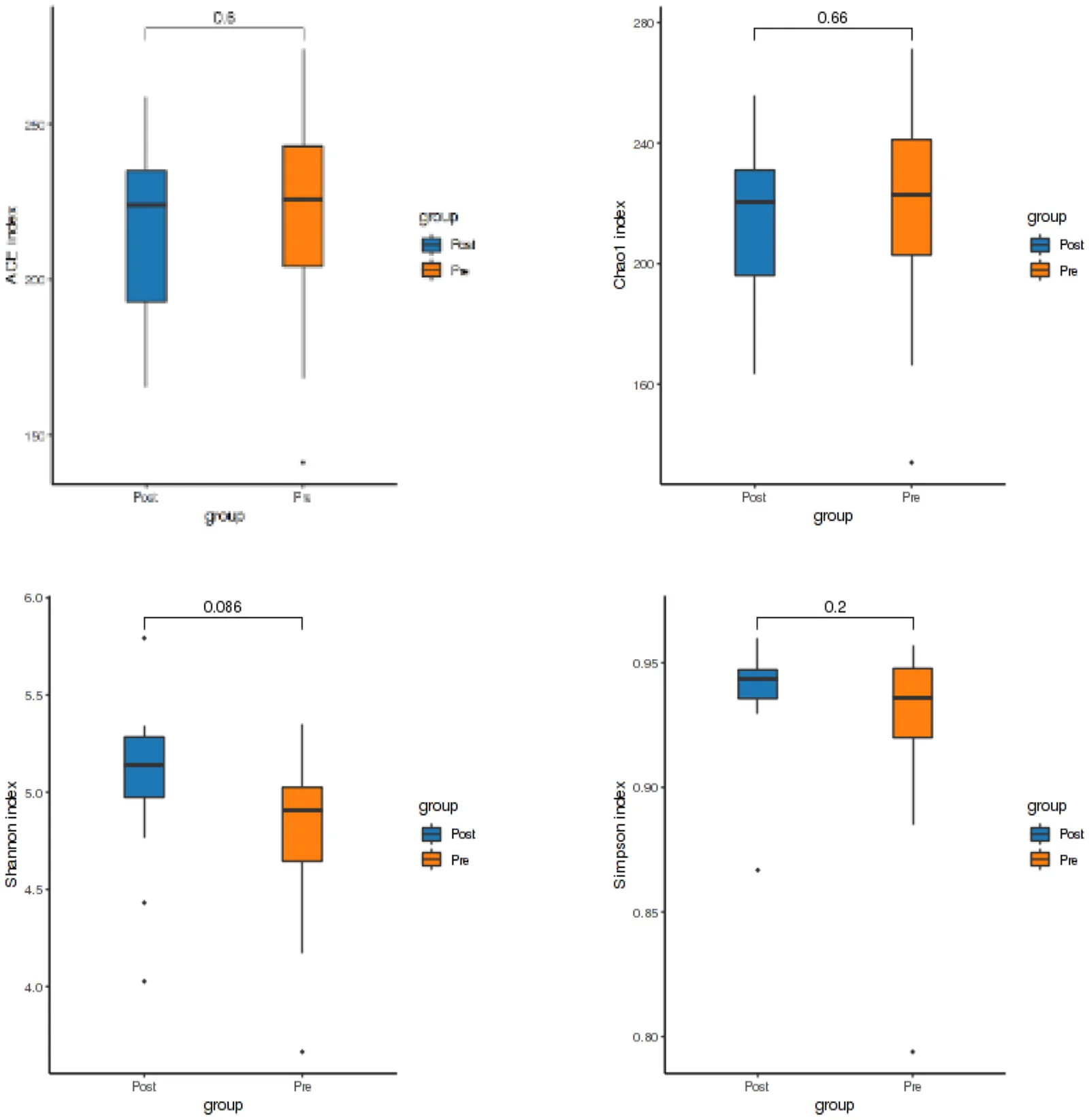
Comparative analysis of alpha diversity between the preoperative pre group and the post group.

As shown in Figure 5, there was no significant difference in the median values of alpha-diversity indices for the oral microbiome between preoperative and postoperative samples in the surgical patients (*P* > 0.05).

#### 3.4.3 Changes in beta-diversity

**Figure 6:**
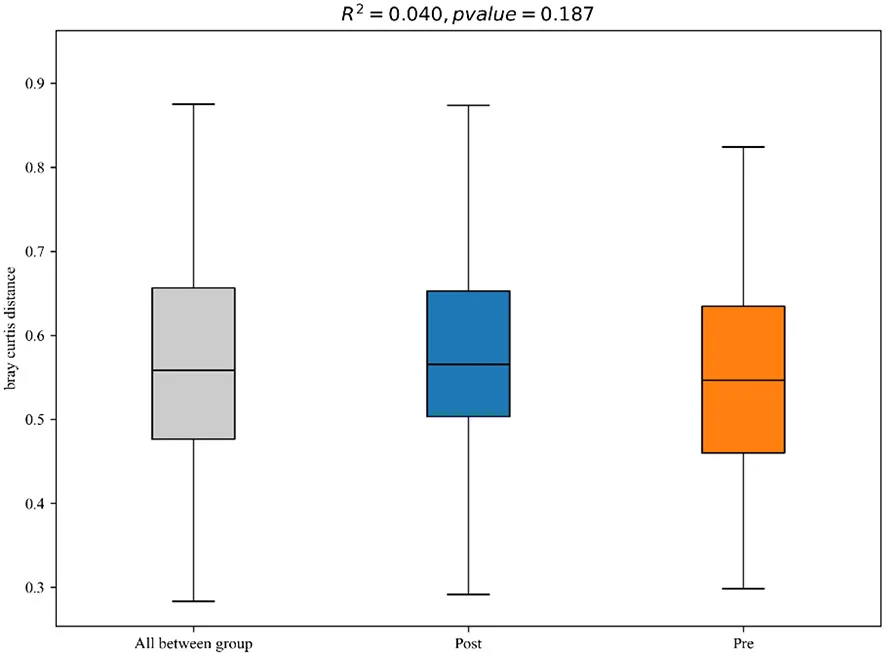
Paired PERMANOVA analysis of pre- and post-operative samples.

**Figure 7:**
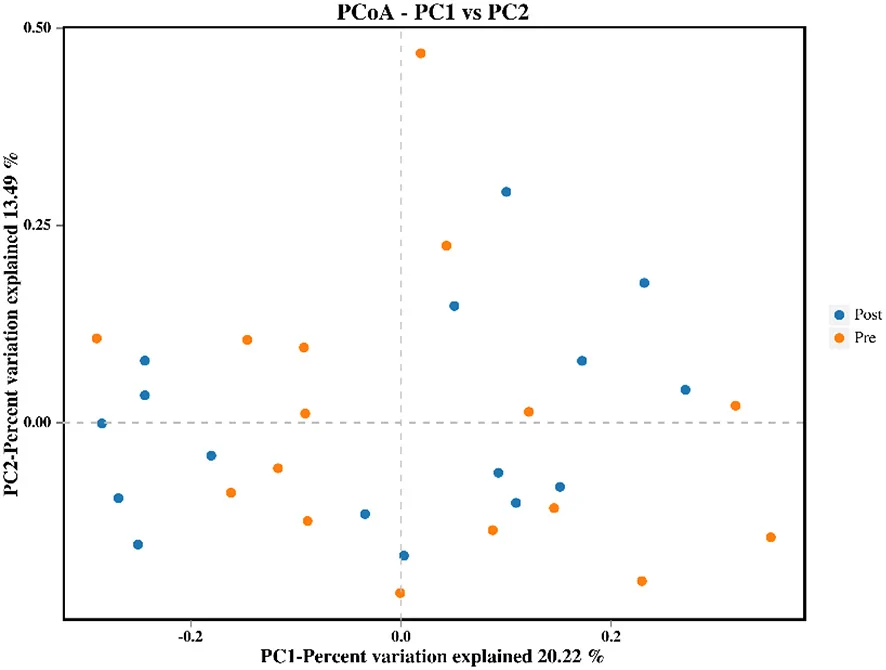
Comparison of P-CoA levels pre- vs. post-operation.

To investigate the macro-level impact of surgery on the overall structure of the oral microbiota, a paired PERMANOVA analysis based on Bray-Curtis distance was performed (Figure 6). The results indicated that the difference in overall microbial community structure between preoperative and postoperative samples did not reach the threshold for statistical significance (*P* = 0.187). In the Principal Coordinates Analysis plot, the postoperative sample points for most patients did not diverge substantially from their own preoperative points (Figure 7). This suggests that no major restructuring of the oral microbial community was detectable at the genus-level compositional scale within the 3-month postoperative observation period.

#### 3.4.4 Changes in abundance of specific taxa

**Figure 8:**
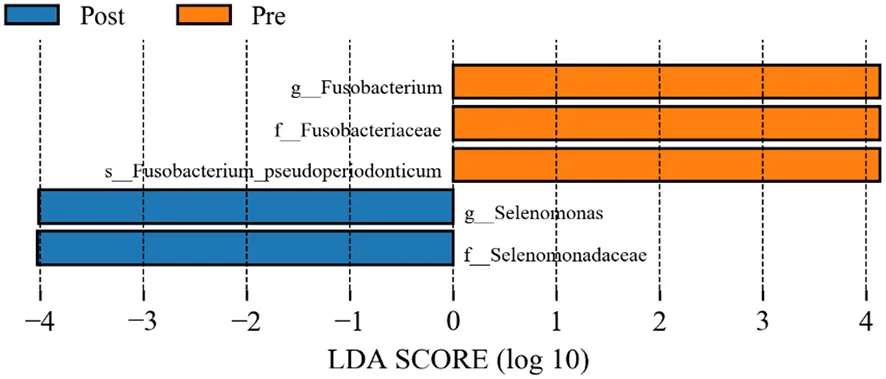
LEfSe analysis to identify differential microbial taxa between pre- and post-operative states.

Although the overall structure remained stable, paired LEfSe analysis (LDA score >4) was performed to identify specific bacterial taxa that underwent significant changes following surgical intervention. The analysis revealed a distinct biomarker shift (Figure 8): the genus *Fusobacterium* was significantly enriched in preoperative samples, whereas the genus *Selenomonas* emerged as the most discriminatory biomarker in postoperative samples. This finding indicates that, against a backdrop of relative structural stability, the surgical intervention selectively altered the relative abundance of key species, inducing a significant biomarker succession within the microbiota.

#### 3.4.5 Dynamic analysis of key cross-sectional genera and emerging postoperative biomarkers

We further analyzed the longitudinal changes in the relative abundance of key genera associated with clinical phenotypes in the cross-sectional study following surgery. The Wilcoxon signed-rank test showed no statistically significant changes in the relative abundances of these genera between the preoperative and postoperative states (all *P* > 0.05). This indicates that the effect of surgery on the oral microbiome was not broad-spectrum, affecting all genera previously identified as altered in the disease state. Instead, the impact was specific, primarily manifested as a reduction in *Fusobacterium* and an increase in *Selenomonas*.

## 4 Discussion

GERD is a highly prevalent digestive disorder with heterogeneous clinical manifestations, posing continuous challenges for its diagnosis and treatment evaluation. Conventional diagnostic tools, such as questionnaires, endoscopy, and reflux monitoring, each have their limitations (Dent et al., 2010; Shaw et al., 2001; Swidnicka-Siergiejko et al., 2022). The composition of the microbiota has been confirmed to influence local and systemic immune responses (Suhail et al., 2021). A growing body of evidence links the oral microbiome to various chronic human diseases, including inflammatory bowel disease, cardiovascular diseases, cancer, diabetes, and rheumatoid arthritis (Gao et al., 2018; Peng et al., 2022). As part of the oral microbiota, the oral microbiome is second only to the gut in terms of diversity and abundance (Escapa et al., 2018). It is readily accessible *via* saliva or oral tissue sampling, with saliva collection being particularly advantageous due to its non-invasive nature, simplicity, and high patient acceptance. Dysbiosis of the oral microbiome has been implicated in the etiology of conditions like dental caries, periodontitis, and even oral cancer (Costalonga and Herzberg, 2014). However, the characteristics of the oral microbiome in GERD and its dynamic changes following surgical intervention remain a frontier in research (Kawar et al., 2021; Liu et al., 2024). LF is an established surgical treatment for GERD, effectively repairing hiatal hernias while providing anti-reflux therapy, with success rates reaching 95% (Frazzoni et al., 2014; Schwameis et al., 2020), yet the pathogenesis of GERD is not fully elucidated.

Integrating cross-sectional and longitudinal study designs, this work systematically investigated the association between GERD and the oral microbiome, and for the first time, revealed the longitudinal effects of LF on oral microbial ecology. Our study yielded several insightful findings. First, cross-sectional analysis confirmed the presence of a distinct oral dysbiosis in GERD patients, with specific bacterial genera correlating with symptom severity and mucosal injury. Second, longitudinal analysis demonstrated that while LF did not cause a global restructuring of the overall microbiome, it triggered a defined ecological succession characterized by the suppression of pro-inflammatory taxa and the proliferation of taxa with potential metabolic benefits.

Initial analysis in this study revealed that, while alpha-diversity of the oral microbiome in GERD patients showed no significant alteration, β-diversity analysis suggested a modest but statistically significant alteration in oral microbial community composition compared with healthy controls. This suggests that the pathological state of GERD, a systemic disease, can be reflected in the specific structural signature of the local oral niche, which may serve as a potential structural biomarker.

More importantly, the differential taxa identified by LEfSe analysis between Group Pre and Group HC hold significant pathophysiological implications. Genera enriched in Group Pre, such as *Alloprevotella* and *Veillonella*, are common opportunistic pathobionts in the oral cavity. *Alloprevotella* can produce endotoxins and inflammatory mediators and has been associated with various chronic inflammatory diseases like periodontitis and rheumatoid arthritis (Koziel and Potempa, 2022). In our study, its relative abundance correlated positively with the DeMeester score, an objective measure of acid exposure. The observed association between *Alloprevotella* abundance and clinical severity should be interpreted with caution. As this was an observational study, the present findings demonstrate statistical associations rather than causal relationships. The enrichment of *Alloprevotella* may reflect alterations in the local oral environment associated with GERD rather than indicating a direct pathogenic role. Likewise, although *Veillonella* abundance was positively associated with GERDQ score, the underlying biological relationship remains to be elucidated through mechanistic and experimental studies.

The core novelty of this study lies in its longitudinal assessment of oral microbiome dynamics following anti-reflux surgery. Paired PERMANOVA analysis indicated no statistically significant shift in overall community structure pre- vs. post-surgery. However, this finding should be interpreted cautiously. Because 16S rRNA amplicon sequencing primarily provides genus-level taxonomic resolution, the absence of significant compositional shifts does not necessarily indicate complete ecological stability at the species, strain, functional, or metabolic levels. Furthermore, the oral cavity is a highly dynamic and environmentally exposed ecosystem influenced by dietary habits, oral hygiene, salivary flow, and host physiological conditions. Therefore, the relatively preserved alpha- and beta-diversity observed in this study more appropriately suggests that no major compositional restructuring was detectable within the resolution and temporal scope of the current analysis, rather than proving absolute stability of the oral microenvironment. Additionally, the 3-month postoperative follow-up period may be insufficient to capture long-term ecological succession following restoration of reflux barrier function. Longer-term longitudinal studies integrating metagenomic, metabolomic, and functional analyses will be necessary to more comprehensively characterize postoperative microbial remodeling.

Several biological mechanisms may explain the oral microbial alterations observed in GERD and the ecological remodeling following anti-reflux surgery. First, repeated exposure of the oral cavity and upper aerodigestive tract to acidic or weakly acidic refluxate may alter oral pH, salivary buffering capacity, and nutrient availability, thereby creating selective pressure that favors acid-tolerant bacterial communities. Second, chronic reflux-induced mucosal inflammation may impair epithelial barrier integrity, modify antimicrobial peptide secretion, and influence local immune responses, collectively contributing to shifts in microbial composition. Third, saliva represents a critical regulator of oral microbial homeostasis. Previous studies have suggested that GERD may be associated with alterations in salivary flow rate and bicarbonate concentration, potentially affecting bacterial colonization, biofilm formation, and interspecies competition. Restoration of the anti-reflux barrier by laparoscopic fundoplication is therefore expected to reduce repeated exposure of the oral cavity to refluxate, alleviate chronic mucosal irritation, and partially normalize the oral ecological environment. Consequently, the microbial changes observed after surgery are more likely to represent secondary ecological remodeling following effective reflux control rather than a direct effect of the surgical procedure itself. Nevertheless, these proposed mechanisms remain speculative and require validation through future multi-omics and experimental studies.

The modulatory effect of surgery was evident at a finer taxonomic resolution. Paired pre-postoperative LEfSe analysis (LDA>4) revealed a marked biomarker succession: the preoperatively enriched, pro-inflammatory *Fusobacterium* was supplanted by the postoperatively enriched *Selenomonas*, a genus involved in organic acid metabolism. *Fusobacterium*, a key member of the periodontal pathogenic complex, is well-documented for its role in inflammation and carcinogenesis in diseases like periodontitis and colorectal cancer, mediated through adhesin expression, toxin production, and host immune activation (Rubinstein et al., 2013; Mesa et al., 2022). Its enrichment in GERD patients may reflect a state of chronic low-grade inflammation in the oral microenvironment associated with reflux. By anatomically and functionally restoring the anti-reflux barrier, LF surgery likely reduces the chronic exposure of the upper aerodigestive tract to refluxate, thereby alleviating persistent physical and chemical irritation of the local microenvironment. The microbial alterations observed after surgery may therefore represent secondary ecological remodeling following reflux control, rather than the primary therapeutic mechanism itself. This may deprive taxa like *Fusobacterium* of a favorable niche, resulting in its significant decrease.

Conversely, the postoperative enrichment of *Selenomonas* points toward a healthier ecological direction. This genus is a key player in the oral glycolytic chain, metabolizing organic acids like lactate (Gonçalves et al., 2012). Notably, some *Selenomonas* strains possess nitrate-reducing capability. Following dietary nitrate intake, they can reduce nitrate to nitrite, a critical precursor in the nitric oxide (NO) generation pathway (Brown, 2025; Latham et al., 2016). NO plays a vital role in maintaining gastrointestinal mucosal blood flow, mucus secretion, and mucosal defense (Cho, 2001). Therefore, the proliferation of *Selenomonas* may signify a shift in the oral microecology toward a state with greater metabolic benefits and potential mucosal protective functions.

This study extends the ecological perspective on GERD from the esophagus to the oral cavity, enriching the understanding of its pathogenesis. The identified oral microbial biomarkers hold future promise as non-invasive tools for auxiliary diagnosis and severity assessment of GERD. Furthermore, it provides novel microbiological evidence supporting the benefits of anti-reflux surgery. By adopting a longitudinal design, this study pioneers the assessment of the long-term impact of anti-reflux surgery on the oral microbiome, serving as a model for future research on surgical modulation of microbiota.

*Limitations:* This study has several limitations that should be considered when interpreting the findings. First, the sample size, particularly for the longitudinal cohort, was relatively limited, which may affect the statistical power, generalizability, and depth of subgroup analyses.

Second, as an exploratory observational study, the present design can reveal associations but cannot definitively establish causal relationships between microbial alterations and GERD pathophysiology or surgical outcomes. Although the longitudinal design provides preliminary temporal evidence, residual confounding factors may still influence the observed microbial dynamics.

Third, this study utilized 16S rRNA amplicon sequencing, which primarily provides genus-level taxonomic information and possesses limited quantitative resolution. Therefore, species-level, strain-level, functional, and metabolic alterations could not be comprehensively characterized. Consequently, functional interpretations regarding microbial metabolism or host-microbe interactions should be regarded as preliminary and hypothesis-generating.

In addition, microbial ecological interaction networks and functional pathway analyses were not incorporated into the present study. While these approaches may provide deeper ecological and mechanistic insights, we considered that the current sample size, particularly in the postoperative cohort, might limit the robustness and interpretability of such analyses.

Finally, although saliva samples were collected using a standardized protocol, factors such as oral hygiene practices, dietary habits, and other lifestyle-related variables could not be completely controlled during follow-up and may have influenced the oral microbial composition. These potential residual confounders should be addressed in future large-scale prospective studies.

*Study novelty:* The present study possesses several important novel aspects. First, unlike previous studies that primarily described taxonomic differences, we integrated oral microbiome profiling with objective clinical phenotypes, including GERDQ score, DeMeester score, and Los Angeles classification, thereby providing clinically relevant evidence regarding microbiota–phenotype associations. Second, by combining cross-sectional and longitudinal study designs, we characterized not only disease-associated microbial alterations but also their dynamic changes following therapeutic intervention. Third, to our knowledge, this is among the first studies to investigate oral microbiome remodeling after laparoscopic fundoplication, providing preliminary evidence that successful restoration of the anti-reflux barrier is accompanied by targeted ecological shifts within the oral microbiome. These findings broaden the current understanding of host–microbiome interactions in GERD and establish a foundation for future mechanistic and translational studies.

*Future directions:* Future investigations should prioritize multi-center studies with larger sample sizes, extended postoperative follow-up, and integration of multi-omics technologies such as metagenomics, metabolomics, quantitative PCR validation, and microbial ecological network analyses to more comprehensively elucidate the mechanistic significance and long-term dynamics of oral microbiota alterations in GERD.

## 5 Conclusion

In conclusion, this study demonstrates that GERD is associated with a distinct oral dysbiosis, marked by an increase in opportunistic pathogens and a decrease in beneficial symbionts. Furthermore, the abundance of specific oral bacteria correlates with objective measures of disease severity, implying their potential involvement in GERD pathogenesis. Importantly, laparoscopic fundoplication was associated with both effective symptomatic improvement and partial ecological remodeling of the oral microbiome toward a healthier compositional profile. These findings provide a preliminary microbiological perspective for understanding the broader physiological impact of anti-reflux surgery and generate hypotheses for future mechanistic investigations.

## Data Availability

The data presented in this study can be found in the NCBI (https://www.ncbi.nlm.nih.gov), accession number PRJNA1510021.
